# Nucleoside analogue 2’-C-methylcytidine inhibits hepatitis E virus replication but antagonizes ribavirin

**DOI:** 10.1007/s00705-017-3444-8

**Published:** 2017-06-16

**Authors:** Changbo Qu, Lei Xu, Yuebang Yin, Maikel P. Peppelenbosch, Qiuwei Pan, Wenshi Wang

**Affiliations:** Department of Gastroenterology and Hepatology, Erasmus MC-University Medical Center, Room Na-621 ‘s Gravendijkwal 230, NL-3015 CE Rotterdam, The Netherlands

## Abstract

Hepatitis E virus (HEV) infection has emerged as a global health issue, but no approved medication is available. The nucleoside analogue 2’-C-methylcytidine (2CMC), a viral polymerase inhibitor, has been shown to inhibit infection with a variety of viruses, including hepatitis C virus (HCV). Here, we report that 2CMC significantly inhibits the replication of HEV in a subgenomic replication model and in a system using a full-length infectious virus. Importantly, long-term treatment with 2CMC did not result in a loss of antiviral potency, indicating a high barrier to drug resistance development. However, the combination of 2CMC with ribavirin, an off-label treatment for HEV, exerts antagonistic effects. Our results indicate that 2CMC serves as a potential antiviral drug against HEV infection.

## Introduction

Hepatitis E virus (HEV) is a single-stranded, positive-sense RNA virus, and its genome contains three open reading frames (ORFs). ORF1 encodes a polyprotein that serves as a precursor of all of the nonstructural proteins needed for HEV replication. ORF2 encodes the capsid protein of the HEV virion. ORF3 encodes a small multifunctional protein with a molecular mass of 13 kDa [11]. HEV was initially thought to cause acute infection only in developing countries. However, over the last decade, hepatitis E cases have frequently been reported in developed countries and have been recognized mainly as autochthonous cases rather than an imported disease [11, 12]. Generally, HEV infection is self-limiting and asymptomatic, and as a consequence, its mortality rate is low. However, it can cause high mortality in pregnant women. In immunocompromised patients receiving organ transplantation, more than 60% of HEV-infected patients develop chronic disease and quickly progress towards severe liver complications such as fibrosis and cirrhosis [20]. In addition to hepatitis, this virus has been associated with a broad range of extrahepatic manifestations, in particular, renal and neurological injuries [14, 21]. Therefore, the development of specific antiviral drugs for HEV infection is urgently required.

Nucleoside analogues have been used clinically for almost 50 years and represent the cornerstones for treatment of patients with cancer or viral infection. Ribavirin (RBV) has been used as an off-label antiviral drug, showing high efficacy in many chronic HEV patients, but HEV mutations associated with ribavirin treatment failure have been reported [4, 7]. Sofosbuvir (SOF), a potent direct-acting agent (DAA) against hepatitis C virus (HCV) [2], has been recently suggested to inhibit HEV replication in cell culture and exert an additive effect when combined with ribavirin [4]. However, other *in vitro* and clinical studies have demonstrated that sofosbuvir is not very effective against HEV infection [8, 18, 19], suggesting that this drug might not be a promising candidate for the treatment of chronic HEV patients.

2’-C-methylcytidine (2CMC) was initially identified as a competitive inhibitor of the HCV RNA-dependent RNA polymerase (RdRp). In addition to HCV, it has been shown to inhibit the replication of a variety of other viruses (*e.g.*, dengue virus and norovirus) [13, 15]. It also has been reported to inhibit cutthroat trout virus, a non-pathogenic fish virus that is remarkable similar to HEV [6]. In this study, we have demonstrated that 2CMC efficiently inhibits HEV replication and thus is a potential candidate for anti-HEV drug development.

## Materials and methods

### Reagents and antibodies

2CMC, RBV, guanosine triphosphate (GTP) and cytidine 5’-triphosphate (CTP) were purchased from Sigma-Aldrich and were dissolved in dimethyl sulfoxide (DMSO) (Sigma-Aldrich, St Louis, MO). The HEV-specific antibody was purchased from EMD Millipore (MAB8002).

### HEV cell culture models

Multiple cell lines were employed in this study, including human hepatoma cell lines (Huh7 and PLC/PRF/5), a human embryonic kidney cell line (HEK293), a human primary glioblastoma cell line (U87), and a human fetal lung fibroblast cell line (MRC5). Huh7 and U87 cells were kindly provided by Professor Bart Haagmans from the Department of Viroscience, Erasmus Medical Center. The human embryonic kidney 293 cell line, PLC/PRF/5 and MRC5 were originally obtained from ATCC (http://www.atcc.org). These cells were cultured in Dulbecco’s modified Eagle medium (Lonza Biowhittaker, Verviers, Belgium) supplemented with 10% fetal bovine serum, 100 IU of penicillin per ml, and 100 μg of streptomycin per ml. For the full-length HEV model, a plasmid construct containing the full-length HEV genome (Kernow-C1 p6 clone; GenBank accession number JQ679013) was employed to generate HEV genomic RNA using an Ambion mMessage mMachine *in vitro* RNA transcription Kit (Thermo Fisher Scientific Life Sciences) [16]. Huh7, PLC/PRF/5, HEK293, U87 and MRC5 cells were electroporated with full-length HEV genomic RNA to generate consecutive HEV-infected cell models (Huh7-p6, PLC/PRF/5-p6, HEK293-p6, U87-p6 and MRC5-p6). To generate the subgenomic (p6-Luc) HEV model, a plasmid construct containing subgenomic HEV was used. This plasmid has an HEV sequence in which the 5’ portion of HEV ORF2 was replaced with the in-frame *Gaussia princeps* luciferase reporter gene [16]. Huh7, U87 and HEK293 cells were electroporated with HEV subgenomic RNA to generate HEV subgenomic models (Huh7-p6-Luc, U87-p6-Luc, and HEK293-p6-Luc). To normalize nonspecific effects of 2CMC on the luciferase signal, Huh7 cells stably expressing a non-secreted firefly luciferase under the control of the human phosphoglycerate kinase (PGK) promotor (PGK-Luc) were used [18]. In addition, Huh7 cells harboring a subgenomic HCV bicistronic replicon (I389/NS3-3 V/LucUbiNeo-ET) (Huh7-HCV-Luc) were used as positive control of antiviral activity.

### Quantification of HEV replication

For *Gaussia* luciferase, the secreted luciferase activity in the cell culture medium was measured using a BioLux^®^ *Gaussia* Luciferase Flex Assay Kit (New England Biolabs). *Gaussia* luciferase activity was quantified using a LumiStar Optima luminescence counter (BMG LabTech, Offenburg, Germany). For the full-length infectious models (HEV-p6), intracellular viral RNA was quantified. RNA was isolated using a Machery-Nucleo Spin RNA II kit (Bioke, Leiden, The Netherlands) and quantified using a NanoDrop ND-1000 spectrophotometer (Wilmington, DE, USA). cDNA was prepared from total RNA using a cDNA Synthesis Kit (Takara Bio Inc, USA). The HEV RNA level was quantified using a SYBR Green–based real-time PCR assay (Applied Biosystems® SYBR® Green PCR Master Mix, Life Technologies, CA, USA) according to the manufacturer’s instructions. The PCR steps consisted of a 10 min holding stage (95 °C) followed by 40 cycles of 15 s at 95 °C, 30 s at 58 °C, and 30 s at 72 °C. Glyceraldehyde-3-phosphate dehydrogenase (GAPDH) was used as a reference gene to normalize gene expression. Relative gene expression was normalized to GAPDH using the formula 2^−ΔΔCT^ (ΔΔCT = ΔCT_sample_ − ΔCT_control_). The HEV primer sequences were as follows: HEV-F, 5’-ATTGGCCAGAAGTTGGTTTTCAC-3’; HEV-R, 5’-CCGTGGCTATAATTGTGGTCT-3’; GAPDH-F, 5’-TGTCCCCACCCCCAATGTATC-3’; GAPDH-R, 5’CTCCGATGCCTGCTTCACTACCTT-3’.

### MTT assay

The cells were seeded in a 96-well plate, and 10 mM 3-(4,5-dimethylthiazol-2-yl)-2,5 diphenyltetrazolium bromide (MTT) (Sigma) was added to the cells. Subsequently, the cells were incubated at 37 °C with 5% CO_2_ for 3 h. The culture medium was then removed, and 100 μl of DMSO was added to each well. The absorbance of each well was read in a microplate absorbance reader (Bio-Rad, Japan) at wavelength of 490 nm.

### Long-term treatment assay

For the long-term treatment assay of the subgenomic model (Huh7-p6-luc), the cells were seeded into a 96-well plate with 5000 cells per well. The cells of the CTR and 2CMC treatment groups were passaged and seeded with the same number of cells every 3 days (d), and cells incubated with vehicle (non-treatment) or 2CMC (10 µM) were maintained throughout the entire incubation period. For the long-term treatment assay of infectious model (Huh7-p6), the cells were seeded into a 48-well plate with 2 × 10^4^ cells per well. The cells of the CTR or 2CMC treatment groups were passaged and seeded with the same number of cells every 3 days, and cells incubated with vehicle (non-treatment) or 2CMC (10 µM) were maintained throughout the entire incubation period.

### Western blot assay

Cultured cells were lysed in Laemmli sample buffer containing 0.1 M DTT and heated for 5 min at 95 °C, followed by loading onto a 10% sodium dodecyl sulfate polyacrylamide gel and separation by electrophoresis for 90 min at 120 V, after which the proteins were electrophoretically transferred onto a polyvinylidene difluoride membrane (Invitrogen) for 1.5 h with an electric current of 250 mA. Subsequently, the membrane was blocked with a mixture of 2.5 ml of blocking buffer (Odyssey, USA) and 2.5 ml of phosphate-buffered saline containing 0.05% Tween 20. This was followed by overnight incubation with anti-HEV capsid protein primary antibodies (1:1000) at 4 °C. The membrane was then washed three times, followed by incubation for 1 h with goat anti-mouse IRDye-conjugated secondary antibody (Li-COR Biosciences, Lincoln, USA) (1:5000). After washing three times, protein bands were detected using an Odyssey 3.0 Infrared Imaging System.

### IC_50_ and CC_50_ calculation

The 50% inhibitory concentration (IC_50_) value and 50% cytotoxic concentration (CC_50_) were calculated based on the model Y¼Bottom þ (Top-Bottom)/ (1 þ 10^((LogIC50-X)*HillSlope)) using GraphPad Prism 5 software (GraphPad Prism 5; GraphPad Software Inc., La Jolla, CA, USA).

### Statistical analysis

Statistical analysis was performed using the nonpaired, nonparametric test with the Mann-Whitney test and one-way ANOVA with Tukey’s multiple comparison post-test (GraphPad Prism version 5.01; GraphPad Software). *P*-values less than 0.05 were considered statistically significant.

## Results

**Fig. 1:**
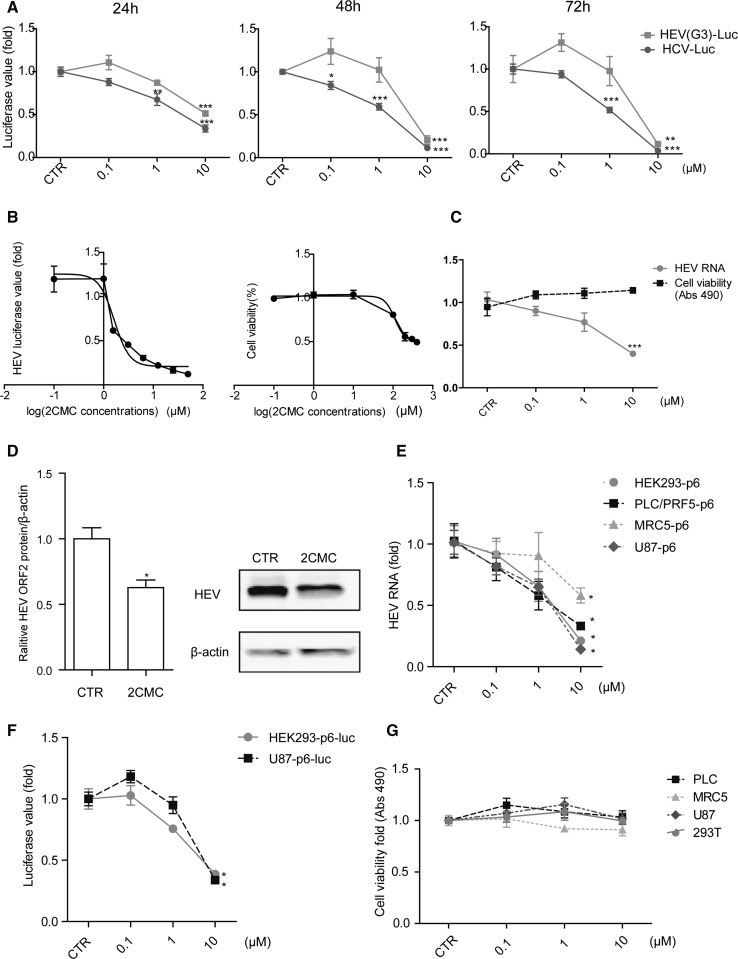
2CMC exerts a potent anti-HEV effect. (A) Huh7-p6-Luc cells and Huh7-HCV-Luc cells were treated with the indicated concentrations of 2CMC for 24 h, 48 h, or 72 h, and the untreated (CTR) group served as a control. Luciferase activity was measured at the indicated time points. Data are the mean ± SEM of four independent experiments. CTR, non-treatment control. *, P < 0.05; **, P < 0.01; ***, P < 0.001. (B) Huh7-p6-Luc cells were treated with 10 μM 2CMC for 48 h. The 50% inhibitory concentration (IC_50_) and 50% cytotoxic concentration (CC_50_) of 2CMC against HEV replication were calculated using GraphPad Prism 5 software. (C) Huh7-p6 cells were treated with the indicated concentrations of 2CMC for 48 h. RT-PCR analysis of HEV RNA and cell viability analysis were performed. Data are the mean ± SEM of four independent experiments. CTR, non-treatment control; Abs 490, absorption at 490 nm; ***, P < 0.001. (D) Immunoblot analysis of the HEV ORF2 protein level in the Huh7-cell-based HEV infectious cell model (Huh7-p6) treated with 2CMC (10 µM) for 48 h. Data are the mean ± SEM of four independent experiments. CTR, non-treatment control; *, P < 0.05. (E) Hepatic and nonhepatic cells were treated with indicated concentrations of 2CMC for 48 h. RT-PCR analysis of HEV RNA was performed. Data are the mean ± SEM of four independent experiments. CTR, non-treatment control. (F) HEK293T-p6-luc and U87-p6-luc cells were treated with the indicated concentrations of 2CMC for 48 h and then were subjected to luciferase activity analysis. Data are the mean ± SEM of three independent experiments. CTR, non-treatment control; *, P < 0.05. (G) The indicated cells were treated with 2CMC for 48 h and then subjected to cell viability analysis using an MTT assay

In this study, the potential anti-HEV effect of 2CMC was investigated in HEV replication models with concentrations ranging from 0.1 μΜ to 10 μM. We demonstrated that 2CMC significantly reduced HEV-driven luciferase activity, and the anti-HEV activity was comparable with its anti-HCV effect at the concentration of 10 µM (Fig. 1A). The IC_50_ value of 2CMC against HEV replication was 1.64 µM, the CC_50_ of 2CMC in Huh7 cells was 111.2 µM, and the selectivity index (SI, CC50/IC50) was 67.8 (Fig. 1B). The anti-HEV effect of 2CMC was further confirmed in the full-length (Kernow-C1, p6) infectious model of HEV genotype 3 by both RT-PCR assay (Fig. 1C) and western blot assay (Fig. 1D).

Since HEV-related extrahepatic manifestations have been reported [12], we extended our study to some other hepatic and nonhepatic cell lines. HEV infectious or replication models were established in HEK293, PLC/PRF/5, MRC5 and U87 cells. The anti-HEV potential of 2CMC in these cell lines was tested. In line with the results observed in Huh7-based HEV replication and infectious models, we observed a similar anti-HEV effect of 2CMC in all these cell models without affecting the cell viability (Fig. 1E to G).

**Fig. 2:**
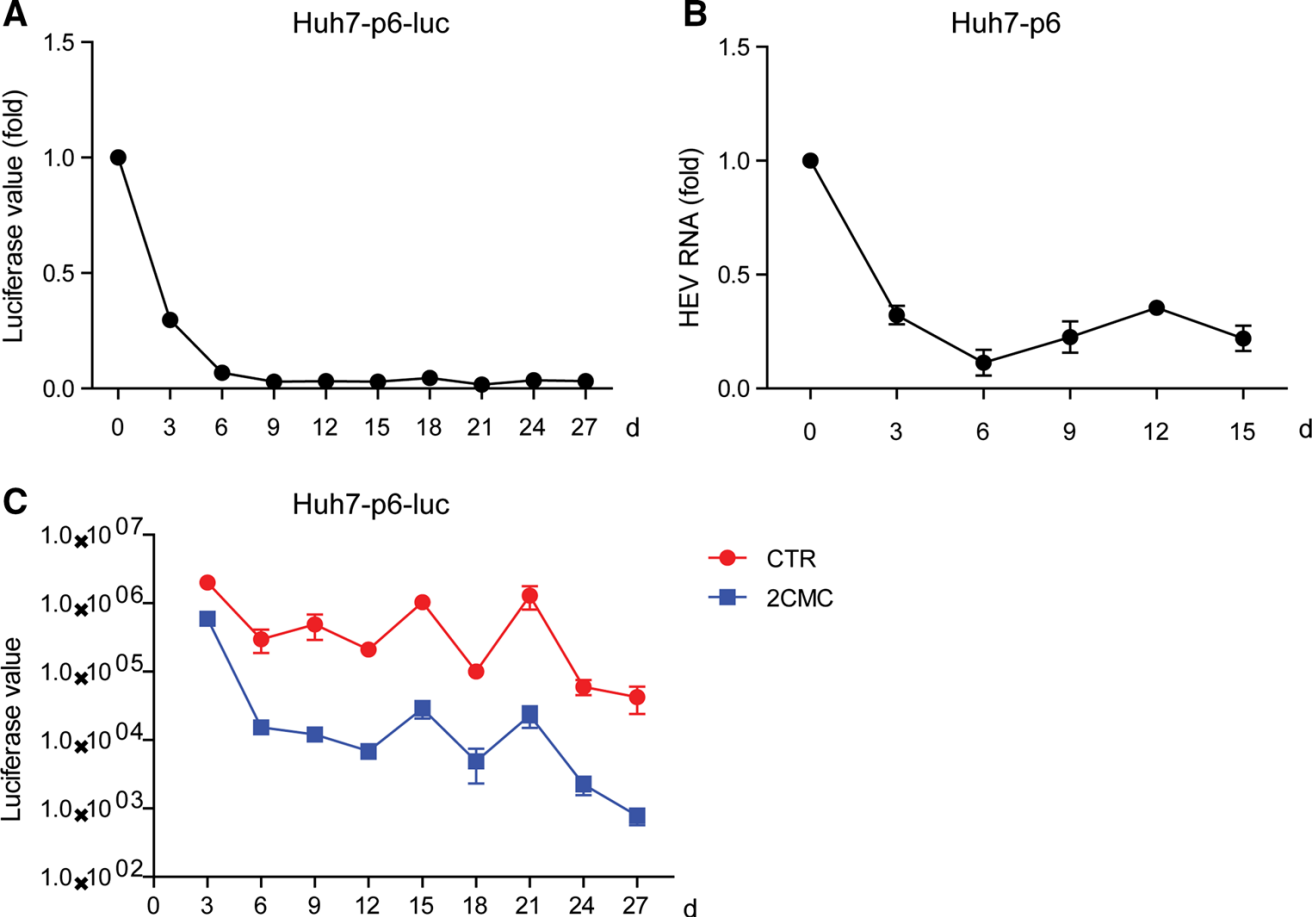
2CMC retains anti-HEV effect in Huh7-p6-luc and Huh7-p6 models after long-term treatment. (A) Treatment of 2CMC in the Huh7-p6-luc model for 27 days. The cells were passaged every 3 days and were incubated with vehicle (non-treatment) or 2CMC (10 μM) throughout the entire period. Data are the mean ± SEM of four independent experiments. CTR, non-treatment control. (B) Treatment of 2CMC in the Huh7-p6 model for 15 days. The cells were passaged every 3 days and were incubated with vehicle (non-treatment) or 2CMC (10 μM) throughout the entire period. Data are the mean ± SEM of four independent experiments. (C) Treatment of 2CMC in the Huh7-p6-luc model for 27 days. The absolute luciferase values of Huh7-p6-luc cells are shown at indicated time points

Drug resistance is one of the main factors that limit the effectiveness of antiviral treatment. To characterize 2CMC in this respect, we performed experiments in which both HEV replication and infectious models were constantly exposed to 2CMC (10 μM). Interestingly, 2CMC retained its anti-HEV activity in both models even after long-term exposure (Fig. 2A and B). Furthermore, the negative control retained high levels of luciferase activity after long-term incubation with 2CMC, excluding the loss of cell viability during the experimental period (Fig. 2C). Taken together, 2CMC displays a high barrier for drug resistance development.

**Fig. 3:**
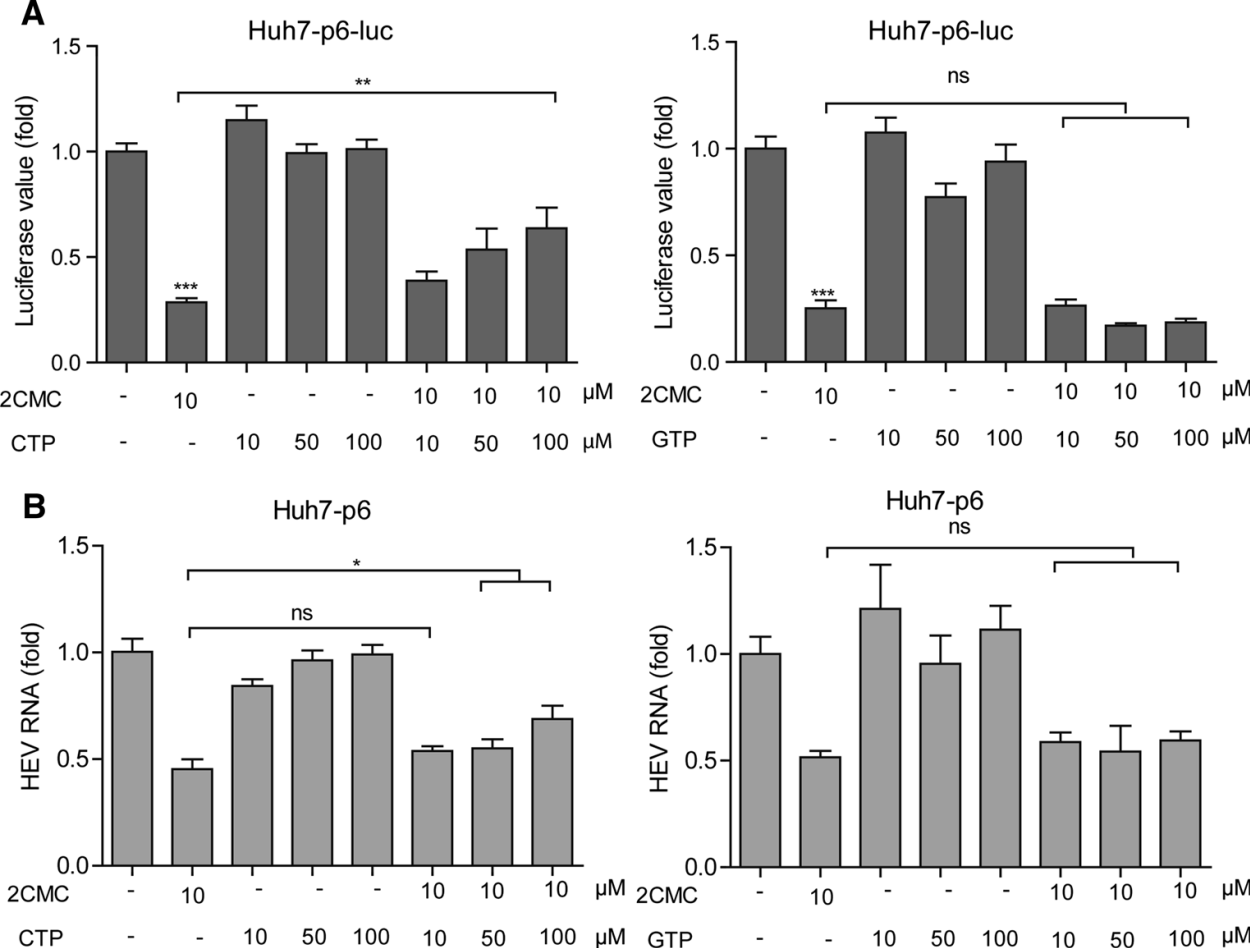
Combination of CTP and GTP with 2CMC in the Huh7-p6-luc model (A) and the Huh7-p6 model (B). The cells were treated with 2CMC, CTP or GTP, alone or in combination for 72 h before measurement of luciferase activity. Data are the mean ± SEM of four to six independent experiments. *, P < 0.05; **, P < 0.01; ***, P < 0.001; ns, not significant

**Fig. 4:**
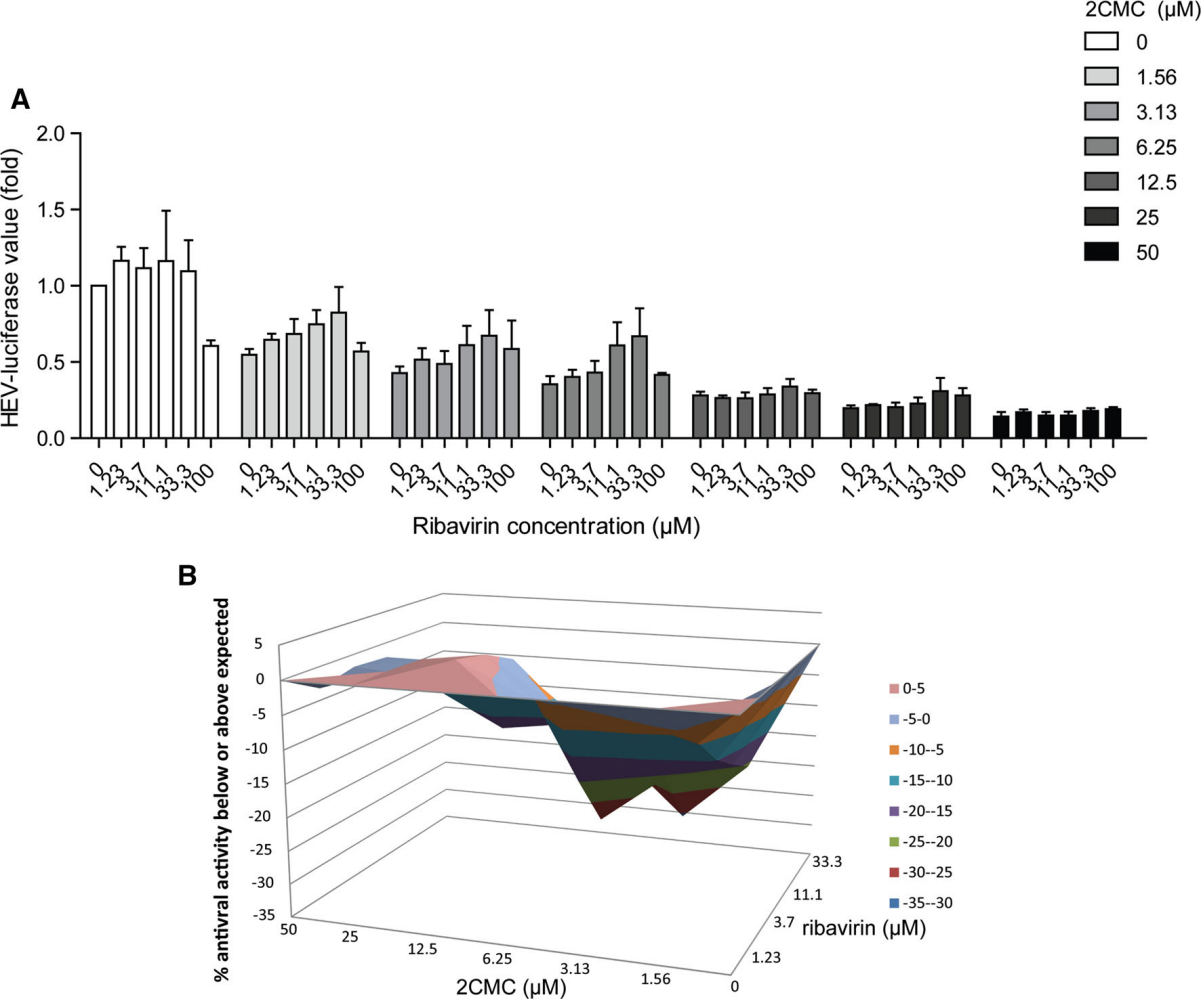
2CMC antagonizes ribavirin in the Huh7-p6-luc model. (A) Huh7-p6-Luc cells were treated with 2CMC and ribavirin, alone or in combination, for 72 h before analysis of luciferase activity. Untreated cells served as a control. (B) The combinatory effect of 2CMC and ribavirin on HEV replication was analyzed using the mathematical model MacSynergy. The three-dimensional surface plot represents the differences (within the 95% confidence interval) between actual experimental effects and theoretical additive effects of the combination at various concentrations of the two compounds. Data are the mean ± SEM of three independent experiments

Theoretically, nucleoside/nucleotide analogs can serve as potential direct-acting antivirals because they bind to the viral RNA polymerase active site to block viral replication. To evaluate the inhibitory specificity of 2CMC against HEV replication, we performed a competition assay employing the substrate cytidine triphosphate (CTP) as an analogous competitor of 2CMC. Our results indicated that CTP reversed the inhibitory effects of 2CMC on HEV replication activity in a dose-dependent manner. In contrast, guanosine triphosphate (GTP) exerted no effect, implying the inhibitory specificity of 2CMC against HEV replication (Fig. 3A and B). Another nucleoside analogue, ribavirin, has been used clinically as an off-label treatment for HEV infection. Thus, its anti-HEV effect in combination with 2CMC was tested. Interestingly, a moderate antagonistic effect (-36.93 µM^2^ %) was observed, implying that they employ a similar antiviral mechanism (Fig. 4A and B).

## Discussion

A variety of nucleoside analogues have been widely used to treat viral infections due to their potent antiviral effects and high barrier to drug resistance development. Ribavirin, an guanosine analogue, is the drug of choice for treating most chronic HEV patients. However, treatment failure has been observed in some cases. Sofosbuvir, a prodrug of a uridine nucleoside analogue that is very effective against HCV, has been investigated recently for its anti-HEV potency. However, there has been debate regarding its potency against HEV [9, 17]. 2CMC, a cytidine nucleoside analogue, has been shown to inhibit infection with a variety of viruses, including HCV and HIV [5]. In this study, we have demonstrated that 2CMC potently inhibits HEV replication in different cell models, albeit with slight differences (Fig. 1A and C). A possible explanation is that these models recapitulate the different steps of the HEV life cycle. The full-length infectious clone (Huh7-p6) models the entire cycle of HEV infection, whereas the subgenomic model (Huh7-p6-luc) only mimics viral replication due to the lack of ORF2 and ORF3.

Encouragingly, the anti-HEV activity was comparable with its anti-HCV effect at particular concentrations. More importantly, in the long-term treatment experiment, 2CMC displayed a high barrier to resistance development, and we demonstrated that this was a specific anti-HEV effect and not due to cytotoxicity. It has been suggested that after it is absorbed by the cells, 2CMC is converted to its 5′-triphosphate form (2CMC-CTP), which serves as an active molecule that competes with the natural substrate CTP. Consistent with this, our results demonstrated that CTP but not GTP reverses the anti-HEV effect of 2CMC, revealing a potential mechanism of action of 2CMC against HEV.

Since ribavirin has been widely used to treat chronic HEV patients, a combined therapy of ribavirin with 2CMC might be envisaged. To test this, the combinatory effects of both drugs were investigated. Unexpectedly, an antagonistic effect was observed. These findings are in agreement with the earlier observation of the combinatory effects of ribavirin and 2CMC on HCV and HIV [3].

Of note, the potential adverse effects of 2CMC should be carefully evaluated in future studies. The clinical applications of nucleoside analogues have been limited in some cases due to off- target effects. Mitochondrial DNA polymerase is an important unintended target for many nucleoside analogues. It has been reported that a nucleoside analogue containing a 2-C-methyl (2-CM) group can reduce mitochondrial transcription and oxidative phosphorylation, resulting in dysfunction of cell metabolism [1, 10]. Therefore, it is recommended that future anti-HEV drug development efforts should focus on the design of less-toxic agents based on the main chemical structure of 2CMC.

In conclusion, 2CMC exerts potent anti-HEV effects in well-established cell culture models, and serves as a potential backbone for anti-HEV drug design. To achieve better efficacy and fewer side effects, future research is still required for drug optimization based on the chemical structure of 2CMC.
